# Implementation and evaluation of the SPRINT protocol for tight glycaemic control in critically ill patients: a clinical practice change

**DOI:** 10.1186/cc6868

**Published:** 2008-04-16

**Authors:** J Geoffrey Chase, Geoffrey Shaw, Aaron Le Compte, Timothy Lonergan, Michael Willacy, Xing-Wei Wong, Jessica Lin, Thomas Lotz, Dominic Lee, Christopher Hann

**Affiliations:** 1 Department of Mechanical Engineering, University of Canterbury, Clyde Road, Private Bag 4800, Christchurch 8140, New Zealand; 2 Department of Intensive Care, Christchurch Hospital, Christchurch School of Medicine and Health Science, University of Otago, 2 Riccarton Ave, PO Box 4345, Christchurch 8140, New Zealand; 3 Department of Mathematics and Statistics, University of Canterbury, Clyde Road, Private Bag 4800, Christchurch 8140, New Zealand

## Abstract

**Introduction:**

Stress-induced hyperglycaemia is prevalent in critical care. Control of blood glucose levels to within a 4.4 to 6.1 mmol/L range or below 7.75 mmol/L can reduce mortality and improve clinical outcomes. The Specialised Relative Insulin Nutrition Tables (SPRINT) protocol is a simple wheel-based system that modulates insulin and nutritional inputs for tight glycaemic control.

**Methods:**

SPRINT was implemented as a clinical practice change in a general intensive care unit (ICU). The objective of this study was to measure the effect of the SPRINT protocol on glycaemic control and mortality compared with previous ICU control methods. Glycaemic control and mortality outcomes for 371 SPRINT patients with a median Acute Physiology And Chronic Health Evaluation (APACHE) II score of 18 (interquartile range [IQR] 15 to 24) are compared with a 413-patient retrospective cohort with a median APACHE II score of 18 (IQR 15 to 23).

**Results:**

Overall, 53.9% of all measurements were in the 4.4 to 6.1 mmol/L band. Blood glucose concentrations were found to be log-normal and thus log-normal statistics are used throughout to describe the data. The average log-normal glycaemia was 6.0 mmol/L (standard deviation 1.5 mmol/L). Only 9.0% of all measurements were below 4.4 mmol/L, with 3.8% below 4 mmol/L and 0.1% of measurements below 2.2 mmol/L. On SPRINT, 80% more measurements were in the 4.4 to 6.1 mmol/L band and standard deviation of blood glucose was 38% lower compared with the retrospective control. The range and peak of blood glucose were not correlated with mortality for SPRINT patients (*P *>0.30). For ICU length of stay (LoS) of greater than or equal to 3 days, hospital mortality was reduced from 34.1% to 25.4% (-26%) (*P *= 0.05). For ICU LoS of greater than or equal to 4 days, hospital mortality was reduced from 34.3% to 23.5% (-32%) (*P *= 0.02). For ICU LoS of greater than or equal to 5 days, hospital mortality was reduced from 31.9% to 20.6% (-35%) (*P *= 0.02). ICU mortality was also reduced but the *P *value was less than 0.13 for ICU LoS of greater than or equal to 4 and 5 days.

**Conclusion:**

SPRINT achieved a high level of glycaemic control on a severely ill critical cohort population. Reductions in mortality were observed compared with a retrospective hyperglycaemic cohort. Range and peak blood glucose metrics were no longer correlated with mortality outcome under SPRINT.

## Introduction

Hyperglycaemia is prevalent in critical care, even with no prior diabetes [1-4]. Increased secretion of counter-regulatory hormones stimulates endogenous glucose production and increases effective insulin resistance [3,4]. Studies also indicate that high-glucose-content nutritional regimes can exacerbate hyperglycaemia [5-10].

Hyperglycaemia worsens outcomes, increasing the risk of severe infection [11], myocardial infarction [1], and critical illnesses such as polyneuropathy and multiple organ failure [2]. Evidence also exists of significant reductions in other therapies such as ventilator support and renal replacement therapy with aggressive glycaemic control [2,12]. More importantly, van den Berghe and colleagues [2,13,14] and Krinsley [15,16] showed that tight glucose control to limits of 6.1 to 7.75 mmol/L reduced relative intensive care unit (ICU) patient mortality by 18% to 45% for patients with a stay of greater than 3 days. Both sets of studies also showed significant cost savings per patient [17,18]. Finally, two recent reviews showed that tighter control with less variability provides better outcome [19,20].

Regulating blood glucose levels in critical care using simple model-based protocols and insulin alone has been moderately successful [21-25]. However, no model-based method has been clinically tested to a mortality endpoint. In contrast, clinically tested sliding scales and titration-based methods have not always been effective, due to an inability to customise the control to individual patients [26-28]. On the other hand, model-based methods are able to identify evolving patient-specific parameters and tailor therapy appropriately.

The significantly elevated insulin resistance often encountered in broad critical care cohorts challenges the practice of using insulin-only protocols. In the presence of significant insulin resistance, insulin effect saturates at high concentrations of insulin [23,29,30], limiting the achievable glycaemic reductions. Hence, despite the potential, many ICUs do not use fixed protocols or necessarily agree on what constitutes acceptable or desirable glycaemic management and performance [4,12,31-34].

However, tighter glycaemic control is still possible by also controlling the exogenous nutritional inputs exacerbating the original problem [5-10]. Clinical studies that intentionally lowered carbohydrate nutrition have significantly reduced average blood glucose levels without added insulin [5,8,9], and Krishnan and colleagues [10] showed that feeding 33% to 66% of the amount recommended by the American College of Chest Physicians (ACCP) guidelines [35] minimised mortality and hyperglycaemia. The present paper presents the clinical implementation of a protocol, developed from model-based controllers [36,37], that modulates both nutrition and insulin to provide tight glycaemic control together with easy clinical implementation. The protocol is a simple paper wheel-based system (Specialised Relative Insulin Nutrition Tables, or SPRINT) that modulates both insulin and nutritional inputs based on hourly or 2-hourly blood glucose measurements for tight glycaemic control. The objectives of this study were to measure the effect of the SPRINT protocol on glycaemic control compared with previous ICU control methods and to evaluate the effect the implementation of the protocol has had on mortality outcomes.

## Materials and methods

### Protocol

Model-based tight blood glucose control is possible with a validated patient-specific glucose-insulin regulatory system model that captures the fundamental dynamics. Chase and colleagues [21,23,38] and Hann and colleagues [38] used a model that captured the rate of insulin utilisation, insulin losses, and saturation dynamics and that has been validated using retrospective data [38-40], clamp data [41], and several short-term (not longer than 24 hours) clinical control trials [36,37]. The model thus captures the metabolic status of the highly dynamic ICU patient and uses it to provide tight control. However, computational resources are not available in some critical care units for effective computerised control methods, and their complexity can limit easy large-scale implementation required to test overall safety and efficacy. Hence, a simpler paper-based method was developed to mimic this protocol. SPRINT was implemented as a clinical practice change at the Christchurch Hospital Department of Intensive Care in August 2005. Further details on SPRINT, its development, and initial pilot study can be found in [27,28,42].

The entry criterion for the SPRINT protocol was a blood glucose measurement of greater than 8 mmol/L on two occasions during standard patient monitoring, where the 8 mmol/L represents the upper limit of clinically desirable glycaemic control in the Christchurch ICU. Patients were occasionally put on SPRINT at the discretion of the clinician if the blood glucose levels were consistently greater than 7 mmol/L in severe critical illness. Patients were not put on the protocol if they were not expected to remain in the ICU for more than 24 hours. Data were collected for all blood glucose measurements, insulin administered, and nutrition given to the patient. The Upper South Regional Ethics Committee, New Zealand, granted ethics approval for the audit, analysis, and publication of these data.

Hourly blood glucose measurements are used to ensure tight control [27]. Two-hourly measurements are used when the patient is stable, defined as three consecutive 1-hourly measurements in the 4.0 to 6.0 mmol/L band [27,42], or when an arterial line is not present. SPRINT is stopped when the patient is adequately self-regulating, defined as 6 or more hours (three 2-hourly measurements) in the 4.0 to 6.0 mmol/L band with over 80% of the goal feed rate and a maximum of 2 U/hour of insulin [27,42].

Total insulin prescribed by SPRINT is limited to 6 U/hour to minimise saturation and the administration of ineffective insulin [23,29,30,43]. Insulin is given predominantly in bolus form for safety, avoiding infusions being left on at levels inappropriate for evolving patient condition. Occasionally, doctors prescribed a background insulin infusion rate of 0.5 to 2 U/hour, primarily for patients known to have type II diabetes, and the insulin bolus recommendations from SPRINT were added to this background rate. A background rate of 0.5 to 1.0 U/hour, to which SPRINT bolus insulin is added, is mandated in patients with type I diabetes.

Goal enteral nutrition rates are approximately 25 kcal/kg per day of RESOURCE Diabetic (Novartis Medical Nutrition, Minneapolis, MN, USA) or Glucerna (Abbott Laboratories, Abbott Park, IL, USA) with 34% to 36% of calories from carbohydrates [44]. Minimum and maximum nutrition rates are 7.5 to 25 kcal/kg per day, with 2.7 to 9 kcal/kg per day from carbohydrates. Thus, an 80-kg male would receive a maximum of 2,000 kcal/day and a minimum of 600 kcal/day, with 216 to 640 kcal/day from carbohydrates, exceeding the minimum level below which there is an increased risk of bloodstream infections [45]. These guidelines are detailed by Shaw and colleagues [26] and are approximately equivalent to the ACCP guidelines [35].

### Statistical analysis

Baseline variables were compared using the two-tailed Mann-Whitney *U *test or chi-square test. Change in mortality was compared between the SPRINT and historical cohorts by means of the chi-square test. The Mann-Whitney and chi-square tests were used to compare blood glucose metrics between survivors and non-survivors. MINITAB^® ^Release 14.1 (Minitab Inc., State College, PA, USA) was used for statistical comparisons, and for all statistical tests, *P *values of less than 0.05 were considered significant.

Log-normal statistics were used to provide an accurate description of blood glucose control results as negative blood glucose concentrations are not possible and typical distributions of blood glucose measurements are asymmetric and show a skew toward higher concentrations. The design of the protocol was that, for periods outside the ideal target range, short periods of higher blood glucose levels were preferred over hypoglycaemic events. Thus, the distributions for blood glucose are right-skewed and log-normal.

### Cohorts

**Figure 1:**
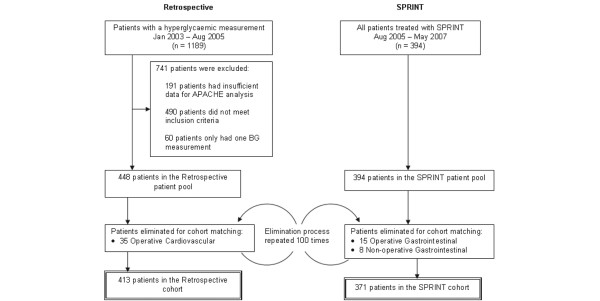
Method of cohort selection for the Specialised Relative Insulin Nutrition Tables (SPRINT) and retrospective patient groups. APACHE, Acute Physiology And Chronic Health Evaluation; BG, blood glucose concentration.

SPRINT was implemented as a clinical practice change and thus was the sole method of treatment for hyperglycaemia. A retrospective cohort has been used to infer changes in patient outcome due to SPRINT. This cohort was extracted from all intensive care patients for the 20-month period of January 2003 to August 2005. Figure 1 shows the selection of patients into the SPRINT and retrospective patient cohorts. Entry criteria into the retrospective cohort were an ICU length of stay of at least 1 day and at least two blood glucose measurements of more than 8 mmol/L spaced not more than 24 hours apart. Patients were excluded where there were insufficient clinical data available to compute an Acute Physiology and Chronic Health Evaluation (APACHE) II score. There was no set protocol for treating hyperglycaemia in the Christchurch ICU during the retrospective period, and clinicians often used a variety of insulin sliding scales.

The retrospective patient pool had a larger proportion of operative cardiovascular patients, and the SPRINT patient pool had a larger proportion of gastrointestinal patients. Changes in the economics of health care caused changes in the types of patients admitted to the Christchurch ICU over the 4-year period encompassed by the SPRINT and retrospective data. The difference in cardiothoracic patients between the patient pools may have resulted from less case throughput and better pre-intensive care glycaemic control. Thus, to provide better-matched cohorts, retrospective operative cardiovascular patients and SPRINT gastrointestinal patients were randomly eliminated from the patient pools to create the cohorts used for analysis, as shown in Figure 1. The patient elimination procedure was repeated 100 times to create 100 cohorts. To present the data clearly, the median cohort results are presented based on mortality outcome for analysis in this article. The major results and outcomes were unaffected by the specific cohort iteration.

## Results

### Patient cohorts

The clinical details of this retrospective cohort are compared with the SPRINT cohort by means of baseline variables, APACHE II scores, and APACHE III diagnosis codes in Table 1.

**Table 1:** Comparison of SPRINT and retrospective cohort baseline variables

	Overall				
	Retrospective		SPRINT		*P *value

Total patients	413		371		
Age, years	64 (53–74)		65 (49–74)		0.53
Percentage of males	59.1%		63.6%		0.19
APACHE II score	18 (15–23)		18 (15–24)		0.50
APACHE II risk of death	28.5% (14.2%-49.7%)		25.7% (13.1%-49.4%)		0.39
Diabetic history	71 (17.2%)		62 (16.7%)		0.86
APACHE III diagnosis					
Operative	Number of patients	Percentage	Number of patients	Percentage	*P *value
Cardiovascular	99	24%	76	20%	0.24
Respiratory	10	2%	9	2%	1.00
Gastrointestinal	53	13%	60	16%	0.18
Neurological	9	2%	7	2%	0.77
Trauma	8	2%	14	4%	0.12
Other (renal, metabolic, orthopaedic)	4	1%	4	1%	0.88
Non-operative	Number of patients	Percentage	Number of patients	Percentage	*P *value
Cardiovascular	41	10%	39	11%	0.79
Respiratory	77	19%	66	18%	0.76
Gastrointestinal	7	2%	10	3%	0.34
Neurological	33	8%	20	5%	0.15
Trauma	29	7%	32	9%	0.40
Sepsis	29	7%	17	5%	0.15
Other (renal, metabolic, orthopaedic)	14	3%	17	5%	0.39

Data are expressed as median (interquartile range) where appropriate. *P *values computed using chi-square and Mann-Whitney *U *tests where appropriate. APACHE, Acute Physiology And Chronic Health Evaluation; SPRINT, Specialised Relative Insulin Nutrition Tables.

### Glycaemic control

Table 2 presents a comparison of glycaemic control for the 371 SPRINT protocol patients against the 413 patients from the retrospective cohort. Measurements (27,664) were recorded for more than 44,769 hours of patient control on SPRINT compared with 13,162 measurements for 43,447 recorded hours of retrospective data. Patients on SPRINT had their blood glucose measured every hour during 24% of their time on the protocol and every 2 hours over the remaining 76% where there was improved glycaemic stability. Log-normal mean blood glucose levels in the SPRINT cohort for hourly and 2-hourly measurements were 6.3 mmol/L (standard deviation 1.6 mmol/L) and 5.6 mmol/L (standard deviation 1.1 mmol/L), respectively. The mean time between measurements in the SPRINT cohort was 1 hour 36 minutes compared with 3 hours 18 minutes for the retrospective cohort. The precision of the recordkeeping system in the Christchurch ICU is to the nearest hour, and nursing staff typically measured blood glucose and used the protocol on the hour.

**Table 2:** Summary comparison of SPRINT and retrospective glycaemic control

Overall cohort data	Retrospective	SPRINT	*P *value
Number of patients	413	371	
Hours of control	43,447	44,769	
Total BG measurements	13,162	27,664	
BG mean (log-normal), mmol/L	7.2	6.0	<0.01
BG standard deviation (log-normal), mmol/L	2.4	1.5	
Percentage of measurements between			
4.4 and 6.1 mmol/L	30.0%	53.9%	<0.01
Percentage of measurements less than			
4.4 mmol/L	6.5%	9.0%	<0.01
4.0 mmol/L	3.8%	3.8%	0.97
2.2 mmol/L	0.2%	0.1%	<0.01
Mean insulin usage, U/hour	1.2	2.8	<0.01
Mean nutrition rate			
During periods of feeding, kcal/day	1,599	1,283	<0.01
Entire duration of SPRINT usage, kcal/day	-	1,014	
Mean percentage of goal feed	-	66.1%	
Per-patient data			
Hours of control	49 (19–140)	53 (19–146)	0.24
Number of BG measurements	15 (6–40)	37 (17–97)	<0.01
BG mean (log-normal), mmol/L	7.4 (6.6–8.3)	6.0 (5.5–6.6)	<0.01
BG standard deviation (log-normal), mmol/L	1.6 (1.2–2.4)	1.3 (1.0–1.8)	<0.01
Percentage of patients <6.1 mmol/L	74.3%	96.0%	<0.01
Insulin usage, U/hour	0.9 (0.1–1.6)	2.6 (2.1–3.3)	<0.01
Nutrition rate			
During periods of feeding, kcal/day	908 (0–1,608)	936 (0–1,308)	0.68
Entire duration of SPRINT usage, kcal/day	-	709 (0–1,167)	
Percentage of goal feed	-	49.7 (0.0–70.8)	

Per-patient data are expressed as median (interquartile range) as appropriate. BG, blood glucose concentration; SPRINT, Specialised Relative Insulin Nutrition Tables.

**Figure 2:**
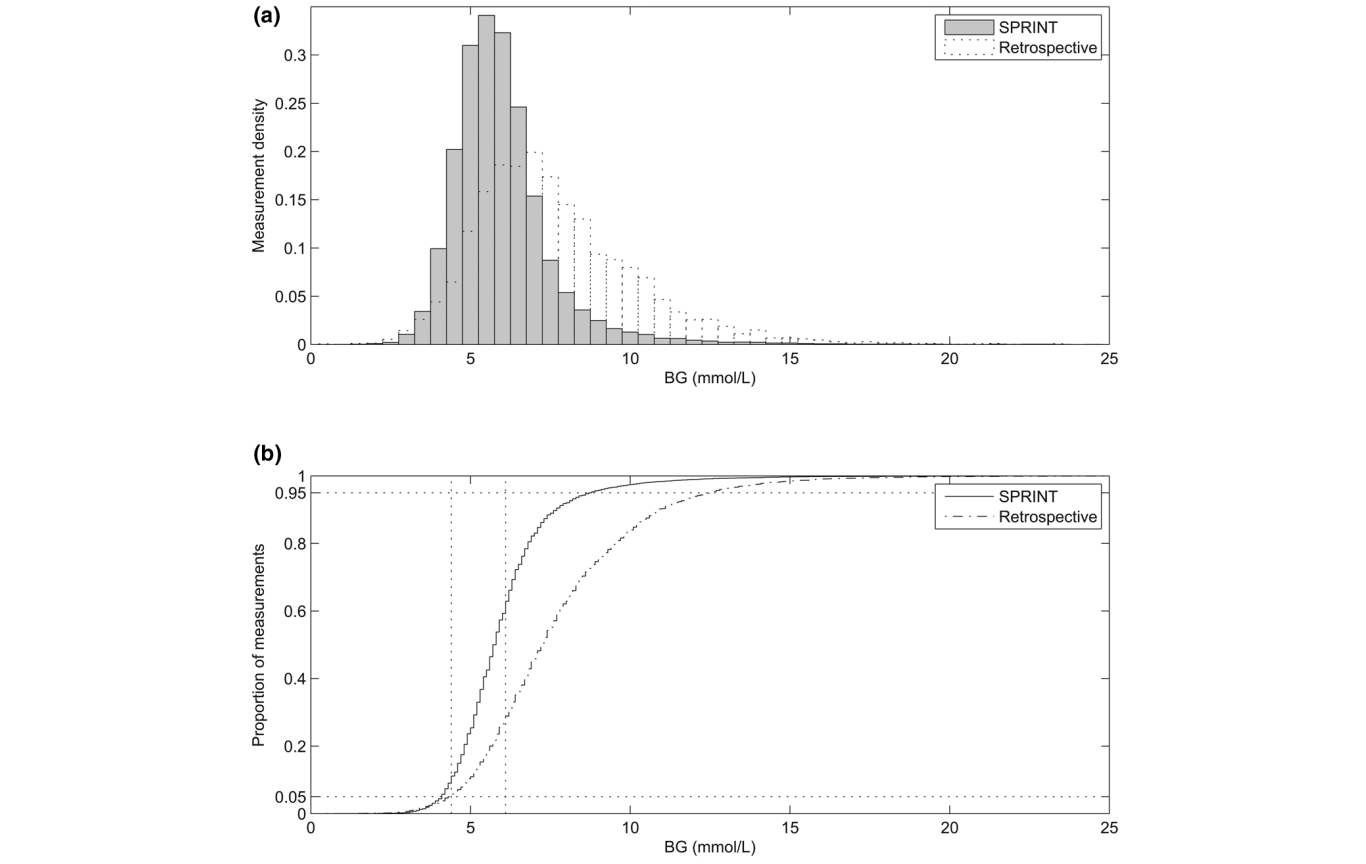
Comparison of distribution of all blood glucose measurements. **(a, b) **Histogram and empirical cumulative distribution function of all blood glucose measurements for all Specialised Relative Insulin Nutrition Tables (SPRINT) patients (shaded, solid line) and retrospective cohort patients (dashed line), respectively. BG, blood glucose concentration.

The percentage time in the 4.4 to 6.1 mmol/L band defined by van den Berghe and colleagues [2,13] was 53.9% compared with 30.0% in the retrospective cohort. Hypoglycaemia was comparable to the retrospective cohort, with only 0.1% of measurements less than 2.2 mmol/L. SPRINT had a higher proportion of measurements below the 4.4 mmol/L limit; however, the two cohorts were comparable for measurements below the 4.0 mmol/L lower limit of the SPRINT target band. Per-patient results show that the mean and standard deviation of blood glucose for SPRINT are lower. Additionally, the interquartile range for both metrics amongst patients is tighter and thus there is less variability in glycaemic control performance between patients. Figure 2 shows a tightly controlled distribution of blood glucose measurements for all patients along with the 4.4–6.1 mmol/L range.

The mean overall hourly insulin usage on SPRINT was 2.8 U/hour, which is a level that avoids insulin saturation effects [29,30,43]. The median feed level recommended by SPRINT was 66.1% of the patient-specific goal feed [42]. The mean overall nutrition rate was 1,283 kcal/day on SPRINT during periods when the patients was being fed, including via the parenteral route, compared with 1,599 kcal/day for the retrospective cohort. The mean nutrition rate over the entire length of stay, including periods in which feed was stopped for reasons outside glycaemic control, was 1,014 kcal/day on SPRINT. When no enteral or parenteral nutrition was recorded in the retrospective cohort data, it was not clear whether the nutrition administration was halted for clinical reasons or because the patient had begun eating meals. Thus, a nutrition comparison with the retrospective cohort was possible only for periods when the patient was receiving enteral or parenteral alimentation.

**Figure 3:**
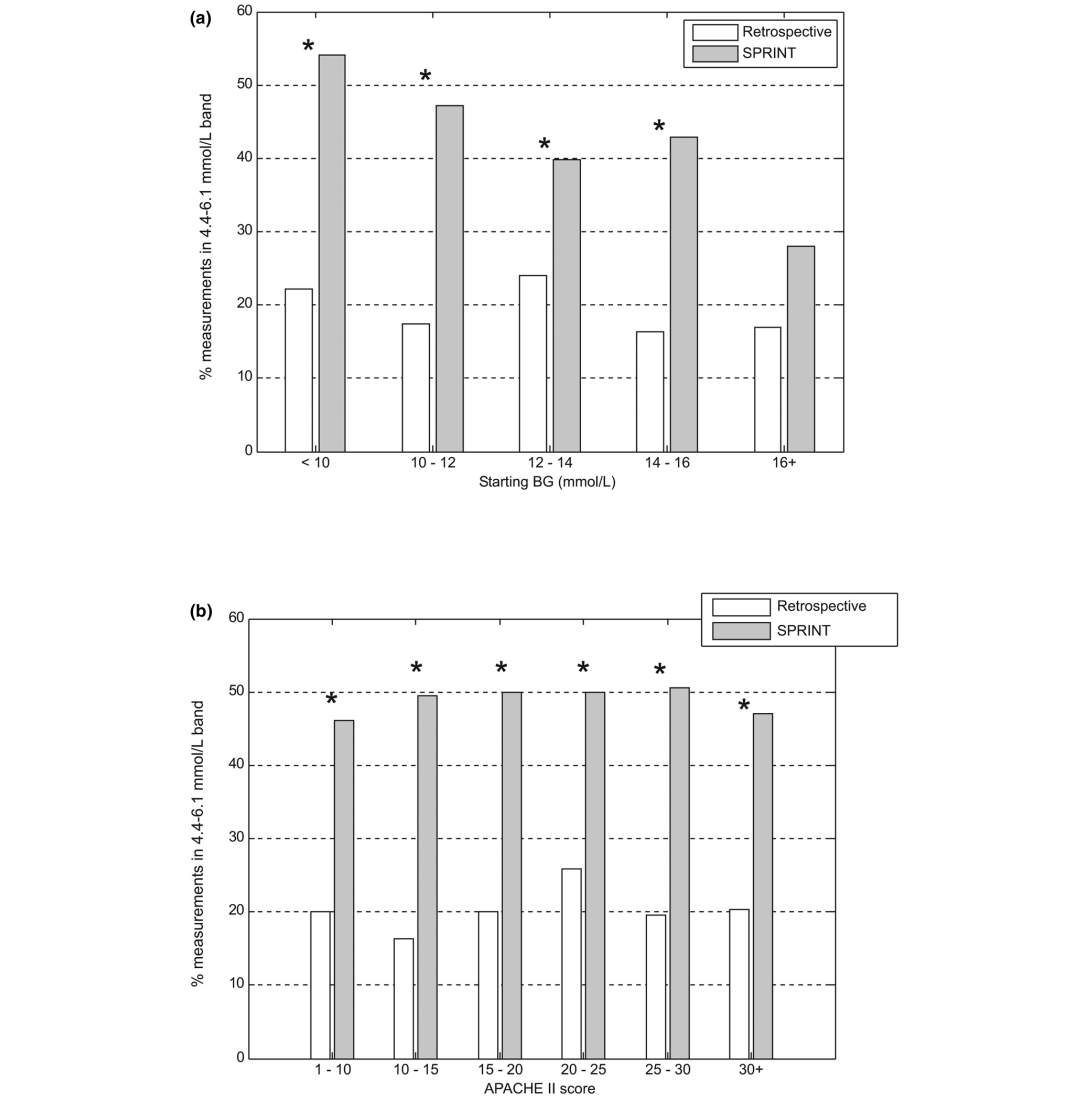
Grouped comparison of percentage of measurements in the 4.4 to 6.1 mmol/L band. **(a) **Measurements grouped by first blood glucose measurement. **(b) **Measurements grouped by Acute Physiology And Chronic Health Evaluation (APACHE) II score. *P < 0.05 (Mann-Whitney test). BG, blood glucose concentration; SPRINT, Specialised Relative Insulin Nutrition Tables.

**Figure 4:**
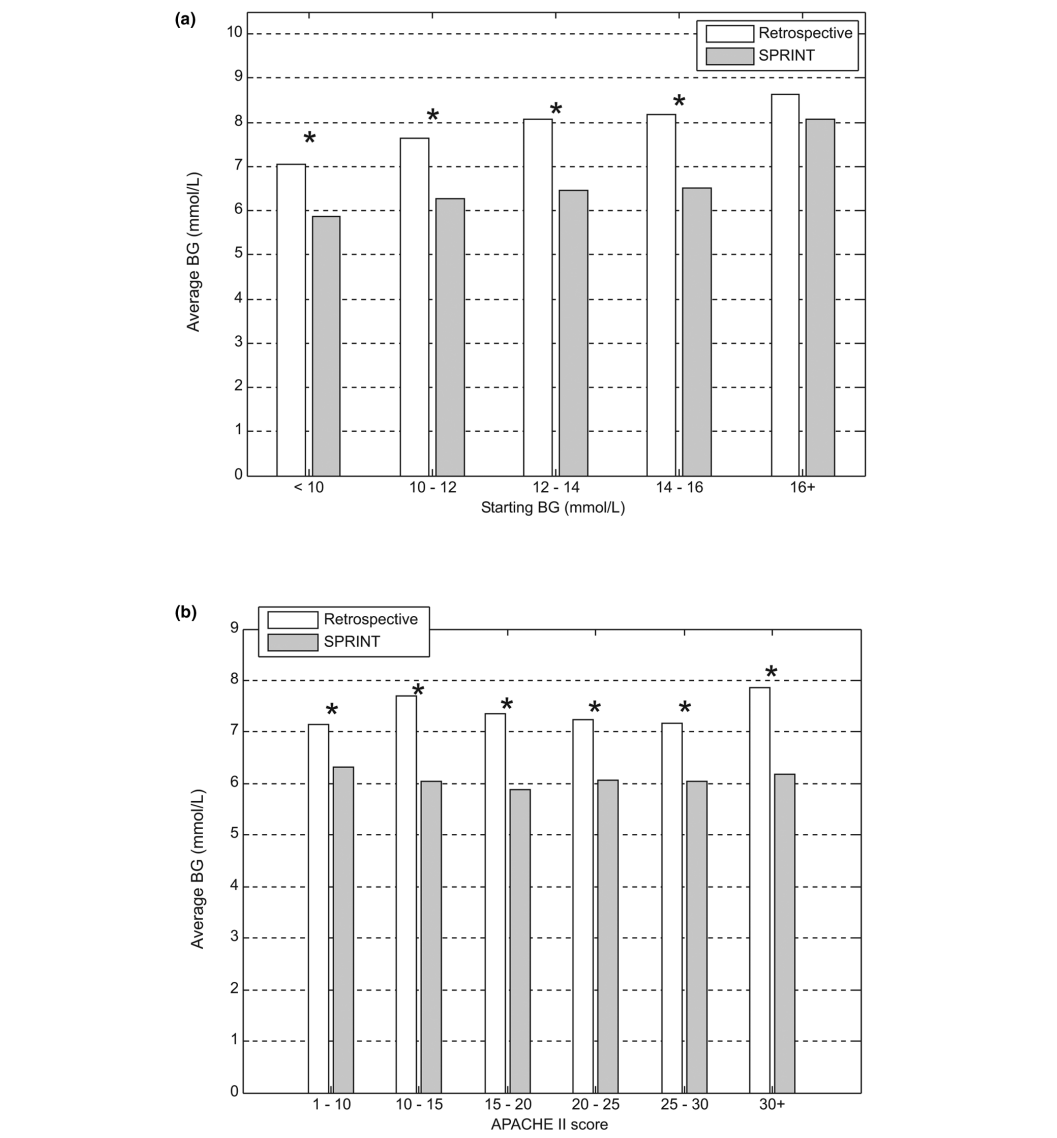
Grouped comparison of average blood glucose level (log-normal). **(a) **Measurements grouped by first blood glucose measurement. **(b) **Measurements grouped by Acute Physiology And Chronic Health Evaluation (APACHE) II score. *P < 0.05 (Mann-Whitney test). BG, blood glucose concentration; SPRINT, Specialised Relative Insulin Nutrition Tables.

**Figure 5:**
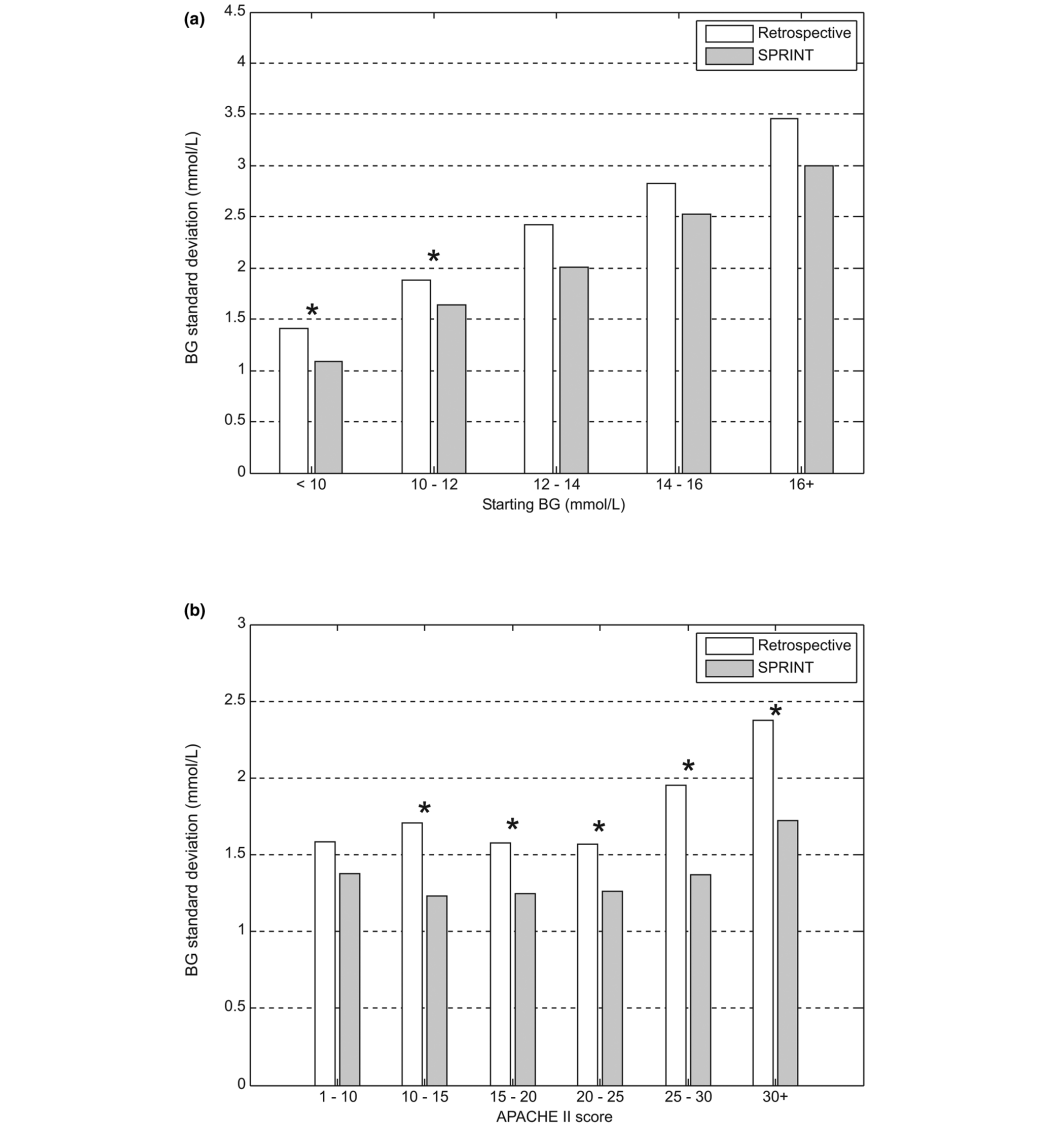
Grouped comparison of blood glucose standard deviation (log-normal). **(a) **Measurements grouped by first blood glucose measurement. **(b) **Measurements grouped by Acute Physiology And Chronic Health Evaluation (APACHE) II score. *P < 0.05 (Mann-Whitney test). BG, blood glucose concentration; SPRINT, Specialised Relative Insulin Nutrition Tables.

Figures 3 to 5 show the average percentage of measurements in the 4.4 to 6.1 mmol/L band, the average blood glucose concentration, and the average blood glucose standard deviation for patients grouped by starting blood glucose level and APACHE II score. The percentage of measurements in the target band was 66% to 203% higher and the blood glucose standard deviation was 6% to 30% lower on SPRINT compared with the retrospective cohort.

**Figure 6:**
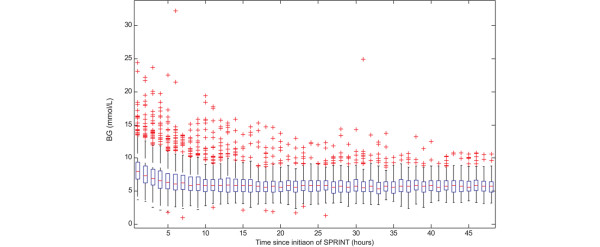
Hourly blood glucose average values for all patients on Specialised Relative Insulin Nutrition Tables (SPRINT). Boxes represent the interquartile range (IQR) containing the median, whiskers represent 1.5 times the IQR, and crosses represent outlying measurements beyond this range. BG, blood glucose concentration.

Figure 6 shows the box-and-whisker plot of hourly blood glucose concentration for all patients over first 48 hours on SPRINT. After approximately 7 hours, the blood glucose median and spread reach their average levels. This level of control is essentially maintained for the remainder of the period. Table 3 shows that 96% of SPRINT patients reached the 6.1 mmol/L band from the initial hyperglycaemic state compared with only 74% of the retrospective hyperglycaemic patients. SPRINT, therefore, brings a patient under control within 7 to 8 hours and maintains a constant level of performance.

**Table 3:** Significance of mortality difference between SPRINT and retrospective cohorts grouped by length of intensive care unit stay

	Intensive care unit mortality			
	Retrospective	SPRINT	Percentage change (relative)	*P *value

LOS ≥ 1 day	18.6%	16.8%	-10%	0.523
LOS ≥ 2 days	20.7%	17.9%	-14%	0.403
LOS ≥ 3 days	22.0%	16.9%	-23%	0.177
LOS ≥ 4 days	22.7%	16.6%	-27%	0.130
LOS ≥ 5 days	21.1%	13.9%	-34%	0.087

	Hospital mortality			

	Retrospective	SPRINT	Percentage change (relative)	*P *value

LOS ≥ 1 day	27.4%	24.9%	-9%	0.457
LOS ≥ 2 days	31.6%	26.3%	-17%	0.170
LOS ≥ 3 days	34.1%	25.4%	-26%	0.045
LOS ≥ 4 days	34.3%	23.5%	-32%	0.020
LOS ≥ 5 days	31.9%	20.6%	-35%	0.019

The *P *values test the median mortality result using the chi-square contingency table test. LOS, length of stay; SPRINT, Specialised Relative Insulin Nutrition Tables.

**Figure 7:**
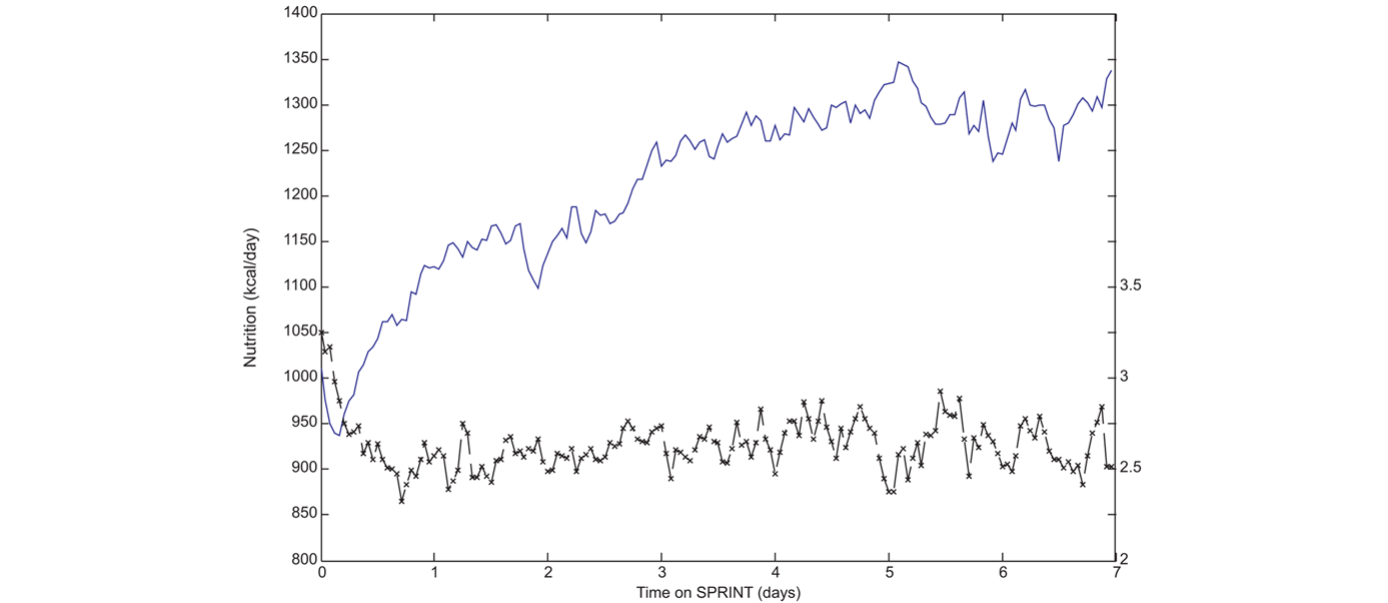
Average nutrition and insulin administration rates for the first 7 days on Specialised Relative Insulin Nutrition Tables (SPRINT). Nutrition rates are represented by solid lines, and insulin rates are represented by dashed lines with crosses.

Figure 7 shows the average nutrition intake and insulin administration rate for the first 7 days on the SPRINT protocol. The average nutrition intake is lower and the average insulin rate is higher during the initial phase of controlling hyperglycaemia. Once hyperglycaemia has been controlled, the average nutrition rate recommended by the protocol increases, generally as patient condition improves and carbohydrate tolerance increases, whilst average insulin administration rate remains relatively constant.

### Clinical outcomes

**Figure 8:**
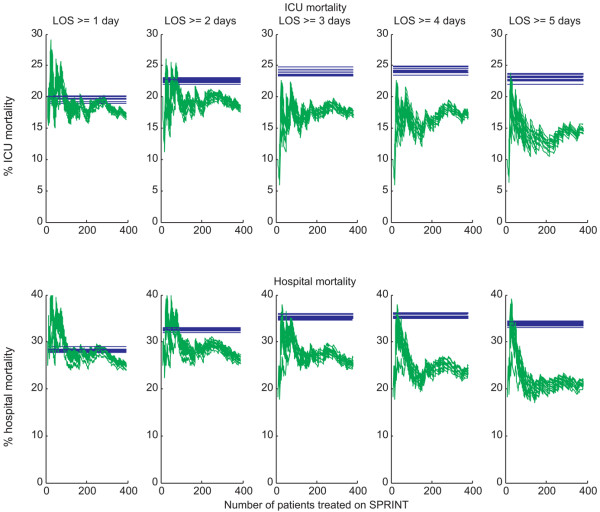
Comparison of intensive care unit (ICU) mortality and in-hospital mortality between Specialised Relative Insulin Nutrition Tables (SPRINT) and retrospective patients for several iterations of the patient selection procedure detailed in Figure 1. The top row of plots shows ICU mortality, and the bottom row of plots shows hospital mortality. The retrospective mortality average is shown by the horizontal line, and mortality since initiation of SPRINT is shown by the variable line. The horizontal axis represents the total number of patients treated with the SPRINT protocol. The plots are grouped by length of ICU stay (LOS). The spread of lines results from the random patient selection process.

Figure 8 shows the percentage mortality for both the SPRINT and retrospective patients for both in-hospital and ICU mortality, grouped by length of ICU stay, for several iterations of the cohort selection procedure described in Figure 1.

Table 3 shows the change in mortality, both in-ICU and in-hospital, for patients with lengths of stay of at least 1 to 5 days, compared with the retrospective cohort using the chi-square test, for the median iteration of the cohort selection procedure. As length of ICU stay increases, the reduction in mortality becomes statistically stronger. Statistical significance (*P *< 0.05) is achieved for an ICU stay of 3 days or longer for in-hospital mortality.

Several recent studies have identified hyperglycaemia as a risk factor for mortality in critical care [1,2,19,46-48]. Table 4 compares average blood glucose, maximum blood glucose, and range of blood glucose between SPRINT ICU survivors and non-survivors by means of the Mann-Whitney test. There is no statistically significant difference between survivors and non-survivors for any of these glycaemic metrics.

**Table 4:** Comparison of statistical significance for patient risk variables between survivors and non-survivors

	Survivors	Non-survivors	*P *value
SPRINT cohort	n = 310	n = 61	
Average BG, mmol/L	6.1 (5.6–6.6)	5.9 (5.5–6.5)	0.55^a^
Maximum BG, mmol/L	9.9 (8.6–11.8)	10.4 (8.5–12.1)	0.62^a^
BG range, mmol/L	6.1 (4.7–8.1)	6.5 (5.0–8.8)	0.35^a^
APACHE II score	18.0 (14.0–22.0)	25.0 (18.0–30.3)	<0.01^a^
APACHE II risk of death	23% (12%-41%)	50% (26%-71%)	<0.01^a^
Average percentage goal feed	48% (0%-71%)	54% (29%-68%)	0.42^a^
Average hourly insulin, U/hour	2.7 (2.2–3.3)	2.5 (2.1–3.2)	0.65^a^
Diabetic history	56 (18.1%)	6 (9.8%)	0.12^b^
Retrospective cohort	n = 336	n = 77	
Average BG, mmol/L	7.5 (6.6–8.4)	7.3 (6.6–8.3)	0.83^a^
Maximum BG, mmol/L	10.5 (9.4–12.6)	11.3 (10.0–13.6)	0.04^a^
BG range, mmol/L	5.8 (3.8–8.6)	6.8 (4.7–9.5)	0.05^a^
APACHE II score	18.0 (15.0–22.0)	24.0 (18.8–29.0)	<0.01^a^
APACHE II risk of death	25% (13%-43%)	51% (23%-67%)	<0.01^a^
Average hourly insulin, U/hour	0.8 (0.0–1.7)	1.0 (0.5–1.5)	0.33^a^
Diabetic history	58 (17.3%)	13 (16.9%)	0.94^b^

Statistical significance was tested for average, maximum and range of blood glucose, APACHE II score, average feed rate, and diabetic history between SPRINT intensive care unit survivors and mortalities. Data are expressed as number (percentage) or per-patient median (interquartile range) where appropriate. ^a^Mann-Whitney *U *test; ^b^chi-square test. APACHE, Acute Physiology And Chronic Health Evaluation; BG, blood glucose concentration; SPRINT, Specialised Relative Insulin Nutrition Tables.

## Discussion

High levels of control were achieved on a patient cohort with relatively severe medical conditions compared with other studies. The median APACHE II score was 18, which is higher than some previous intensive insulin clinical studies whose APACHE II medians or averages were 9 [2,13] and 16.9 [15]. Higher APACHE II scores are a general indicator of increased insulin resistance [15].

The overall mean of 6.0 mmol/L with a standard deviation of 1.5 mmol/L compares well with the 5.7 ± 1.0 mmol/L value of van den Berghe and colleagues [2], who studied a much less ill cohort. Similarly, it is lower than the 7.3 ± 3.4 mmol/L result reported by Krinsley [15] for a less critically ill cohort. Finally, it is similar to the 6.0 ± 1.3 mmol/L reported by van den Berghe and colleagues [14], who reported an average APACHE II score of 24. It is also important to note that van den Berghe and colleagues [14] reported only mean morning glucose, which a recent large study showed to be significantly lower than other daily measurements [49], thus possibly minimising the variation reported.

SPRINT was implemented as a clinical practice change and thus only a comparison with retrospective data was possible. A limitation of this study is that the SPRINT and retrospective groups were comparable only on a cohort-wide basis. However, as shown in Figure 8, there is a strong signal for the reduction in mortality following the introduction of a high-performance glycaemic control protocol. Mortality reductions were statistically stronger for patients who stayed in the ICU for increasingly longer periods. The plots of Figure 8 indicate a trend toward a steady-state reduction in mortality, particularly for patients with longer ICU stays, and show that greater statistical significance may be reached with a larger cohort, which agrees with the results of van den Berghe and colleagues [2].

Figures 2 and 6 show that as mean blood glucose decreases the lower 5% limit of measurements does not appreciably drop, indicating that control is not simply shifting blood glucose lower but is also tightening the spread and thus minimising the risk of hypoglycaemic events. Table 2 shows that 0.1% (n = 24) of measurements were less than or equal to 2.2 mmol/L, with only 20 (5.2%) patients experiencing one or more such measurements. Thus, lower and tighter glycaemia was achieved without increasing the risk of hypoglycaemia.

Time in a relevant glycaemic band can provide a more robust description of control performance than an average glycaemic value. This result is consistent across all starting blood glucose ranges and APACHE II scores shown in Figures 3 to 5 and emphasises the consistency of control achieved under varying patient conditions. Grouping patients by these metrics enables comparisons between more similar groups.

The mean nutrition rate puts the caloric intake of 1,283 kcal/day on SPRINT in the middle tertile of the ACCP guidelines reported by Krishnan and colleagues [10] to be optimal for mortality outcome. The median per-patient average nutrition rates were similar for the SPRINT and the retrospective cohorts.

Table 4 shows that SPRINT has removed glycaemia as a statistically correlated risk factor of ICU mortality. In the retrospective cohort, maximum blood glucose and range of blood glucose were still associated with mortality. Additionally, APACHE II score was significantly higher in non-survivors for both cohorts. The APACHE II risk of death (which depends upon diagnosis) was also higher in non-survivors than survivors, with a median risk of death of 50% versus 23% in non-survivors versus survivors on SPRINT. There was no significant difference in percentage of goal feed rates between survivors and non-survivors for the SPRINT cohort.

A recent review of published insulin-based glycaemic control protocols in intensive care identified protocols that adjusted insulin infusion based on frequently measured changes in blood glucose concentration as providing the best control [50]. SPRINT is derived from a model-based controller that incorporates non-linear insulin transport and glucose removal kinetics. Thus, it was identified in simulation that, in addition to insulin usage, feed modulation is required for ideal control [27]. SPRINT uses the absolute blood glucose level, change in blood glucose level, and current insulin and nutrition administration rates to identify effectively the patient's insulin sensitivity and respond accordingly. The unique wheel-based design of SPRINT allows all of these metrics to be incorporated into an essentially nurse-automated protocol.

The paper-based design of the SPRINT system allowed for relatively easy adoption into the Christchurch ICU, which does not typically have suitable bedside computing resources available. Computerised methods can have several advantages over paper-based systems. The SPRINT wheels are discretised to increments of 1 U of insulin and 10% of the goal feed to provide a design that is compact and easy-to-use, whereas a computer implementation could allow more refined dosing recommendations. Complete electronic recordkeeping is also possible on computerised systems, which can assist in measuring protocol compliance.

SPRINT is fully implemented by nursing staff without additional clinical intervention or modification. We believe that the frequent blood glucose measurement required by SPRINT is justified as the protocol prescribes definitive actions and provides tight safe results. Hence, it outlines what to do under most or all conceivable scenarios, limiting the need for the clinician intervention or modification seen in other protocols.

## Conclusion

SPRINT was implemented as a clinical practice change and achieved an average blood glucose level of 6.0 ± 1.5 mmol/L, with 53.9% of measurements in the 4.4 to 6.1 mmol/L band over a general ICU cohort. Reductions in ICU and hospital mortality rates were observed in comparison with a retrospective cohort. Risk factors such as maximum blood glucose and range of blood glucose were no longer associated with survival under SPRINT.

## Key messages

• High-performance glycaemic control can be achieved with a simple nurse-automated protocol.

• Modulating both insulin and nutrition in tandem can achieve tight consistent glucose control.

• Reductions in mortality for longer-stay (>3 days) patients suggest that tight control improves outcomes.

• The maximum and range of blood glucose measurements were no longer correlated with mortality, due to tight control removing these glucose metrics as indicators of outcome.

• Comparing glucose performance metrics on a per-patient basis can give important information on the variation of protocol performance between patients.

## Abbreviations

ACCP = American College of Chest Physicians; APACHE = Acute Physiology And Chronic Health Evaluation; ICU = intensive care unit; SPRINT = Specialised Relative Insulin Nutrition Tables.

## Competing interests

JGC and GS hold shares in Intersection LifeSciences (Christchurch, New Zealand). JL, T Lonergan, and T Lotz have been employees of Intersection LifeSciences since April 2007. Portions of the SPRINT protocol are patent-pending in the US.

## Authors' contributions

JGC helped conceive and develop the SPRINT protocol and helped draft the manuscript. GS helped conceive and develop the SPRINT protocol and assisted in implementing the protocol in the Christchurch ICU. ALC helped conceive and develop the SPRINT protocol, assisted in data collection and the analysis and interpretation of the data, and helped draft the manuscript. T Lonergan and MW helped conceive and develop the SPRINT protocol. XWW, JL, and T Lotz assisted in data collection and the analysis and interpretation of the data. DL provided statistical assistance. CH provided mathematical assistance during the development of SPRINT. All authors read and approved the final manuscript.
